# A Case of Sheehan Syndrome Six Years Postpartum Presented With Adrenal Crisis and Complicated by Hypothyroidism and Massive Pericardial Effusion

**DOI:** 10.7759/cureus.33972

**Published:** 2023-01-19

**Authors:** Priya Mishra, Himanshu Jindal, Efa Khan, Sandeep S Palawat

**Affiliations:** 1 Department of Internal Medicine, Ganesh Shankar Vidyarthi Memorial Medical College, Kanpur, IND

**Keywords:** sheehan syndrome, postpartum hypopituitarism, pituitary apoplexy, hypopituitarism, empty sella syndrome, adenohypophyseal hyposecretion

## Abstract

Sheehan syndrome is often a sequela of massive postpartum hemorrhage in resource-poor healthcare settings where blood loss during delivery is often neglected. The diagnosis of this rare but fatal disease is often delayed because the symptoms are vague and the pituitary dysfunction is insidious in nature. We report the case of a 35-year-old multiparous female with anhedonia and raised serum transaminases. She presented with constitutional symptoms. Her last vaginal delivery, six years back, was the last obstetric event that yielded a stillbirth child. She had had amenorrhea since then. Upon further evaluation, she was found to have a massive pericardial effusion, hypopituitarism, and a partially empty sella. This case report highlights the uncharacteristic symptoms that a patient presents with which ultimately lead to delayed diagnosis. Early diagnosis can go miles in improving the quality of life of the patient besides saving the patient from an adrenal crisis.

## Introduction

First described by HL Sheehan in 1937, Sheehan syndrome refers to ischemic necrosis of the adenohypophysis due to severe hypotension usually resulting from a massive postpartum hemorrhage (PPH) [1]. It can present acutely or months to years after a delivery complicated by PPH, with symptoms of partial or panhypopituitarism. The majority of Sheehan syndrome cases occur in underdeveloped or developing countries where the risk of obstetric complications is high and skilled obstetric care is scarce, particularly in rural areas.

Certain factors have been linked with the pathogenesis of Sheehan syndrome ­­- pituitary enlargement in pregnancy via estrogen-mediated hyperplasia of lactotrophs. An enlarged pituitary may compress the superior hypophyseal artery, a predisposition to severe ischemia in the case of massive blood loss [1,2].

Clinical presentations vary depending on the deficient pituitary hormones, ranging from selective hormonal insufficiency to classic panhypopituitarism. Magnetic resonance imaging (MRI) of the brain is the diagnostic test of choice, an empty or partially empty sella being the characteristic finding except in acute cases. Some remarkable findings include sparse pubic and axillary hair, dry skin, fine wrinkling around the mouth, and involution of breast and vaginal atrophy. Adrenocortical insufficiency due to corticotroph failure is an important consequence of Sheehan syndrome that can result in hypotension, asthenia, hypopigmentation, hyponatremia, and hypoglycemia [3].

The mean interval between a complicated delivery and diagnosis of Sheehan syndrome is reported to be around 13 years. Delayed diagnosis can be explained by non-specific presentations such as fatigue, anemia, and asthenia during the postpartum period which may be misdiagnosed as having baby blues [4]. Most patients remain asymptomatic for a prolonged duration, their condition may worsen during acute stressors resulting in adrenal crisis, myxoedema, coma, or death. This article was previously posted to the Research Square preprint server on March 23, 2022.

## Case presentation

A 35-year-old female, gravida three para three (G3P3), presented to the emergency medicine department with generalized body weakness for two months, fever for three days, and loss of appetite for 15 days. On admission, the patient was found to be hypotensive (BP: 64/30 mm of Hg) with a pulse rate of 112/min, Glasgow Coma Scale (GCS) of 11 (E_3_V_3_M_5_), and oxygen saturation of 95% on room air. She had menarche at 15 years of age with normal subsequent sexual development. She admitted to having recurrent episodes of jaundice that resolved upon the local practitioner’s intervention. Her history did not reveal hospitalization for any medical condition in the past except for her deliveries. She was on anti-tubercular therapy (ATT) for the past three months. In her obstetrical history, she mentioned two vaginal deliveries that were uneventful. However, her last vaginal delivery (six years back) at a primary health care (PHC) center yielded a stillbirth child and she was kept under observation for two days for unknown reasons. The delivery took place with the help of drugs to augment labor for a vaginal delivery. This was her last delivery. She began losing weight, her health deteriorated and, she had had amenorrhea since then. She gradually had progressive body weakness and anhedonia to the extent that she could not perform day-to-day activities and was essentially bedridden.

**Figure 1:**
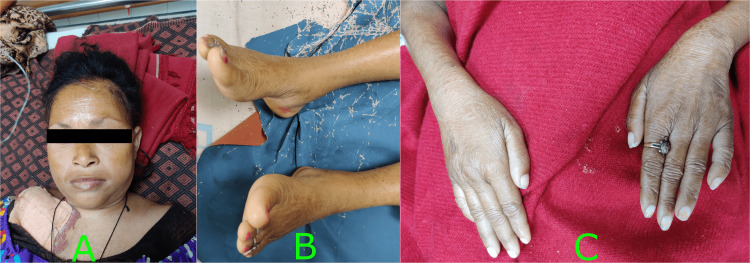
Photographs of the patient showing: (A) Coarse facial features with facial edema, wrinkles, scanty scalp hair on the forehead, and partial loss of both eyebrows; (B) Dry, scaly, and pale skin of legs; (C) Dry and scaly skin of hands

Her physical examination revealed anasarca, generalized pallor, and asthenia. She had thinning limbs, facial edema, hair fall, and dryness of skin (Figure 1).

The CBC (complete blood count) revealed thrombocytopenia (135,000 cells/mm^3^) and erythropenia (3.51 x 106/mm^3^) (Table 1). She was thus transfused with two units of blood on the third day of admission.

**Table 1:** Results of complete blood count (CBC), kidney panel, and liver panel enzymes of the patient MCV: mean corpuscular volume, MCH: mean corpuscular hemoglobin, MCHC: mean corpuscular hemoglobin concentration, SGOT: serum glutamic oxaloacetic transaminase, SGPT: serum glutamic pyruvic transaminase, ALP: alkaline phosphatase

Parameter	Day 1 (On admission)	Day 4	Day 7	Day 14	Reference Range
Hemoglobin, g/dL	6.3	10.5	9.9	10.0	12-16.5
Total leukocyte count, cells/mm^3^	8,700	7,500	6,600	12,100	4,000-10,000
Platelet count, cells/mm^3^	135,000	79,000	61,000	38,000	150,000-450,000
Red blood cells, x 10^6 ^cells/mm^3^	2.91	4.11	4.1	4.22	3.8-4.8
MCV, fL	62.8	62.2	72.1	72.5	80-100
MCH, pg	21.7	25.6	24.1	23.8	27-32
MCHC, g/dL	34.5	41.1	33.5	32.9	32-35
Hematocrit, %	18.2	25.6	29.5	30.6	36-46
Serum urea, mg/dL	44	-	-	46	13-43
Serum creatinine, mg/dL	1.6	-	-	1.2	0.6-1.2
Serum bilirubin (total), mg/dL	3.8	2.7	-	-	0-1.2
Serum bilirubin (direct), mg/dL	1.0	1.5	-	-	0-0.2
Serum bilirubin (indirect), mg/dL	2.8	1.2	-	-	0.2-0.7
Serum protein, g/dL	6.2	6.4	-	5.8	6.0-8.3
Serum albumin, g/dL	3.4	4.2	-	4.0	3.8-5.5
SGOT, IU/L	71	74	-	33	<40
SGPT, IU/L	14	12	-	63	<34
Serum ALP, IU/L	398	406	-	220	<240

**Figure 2:**
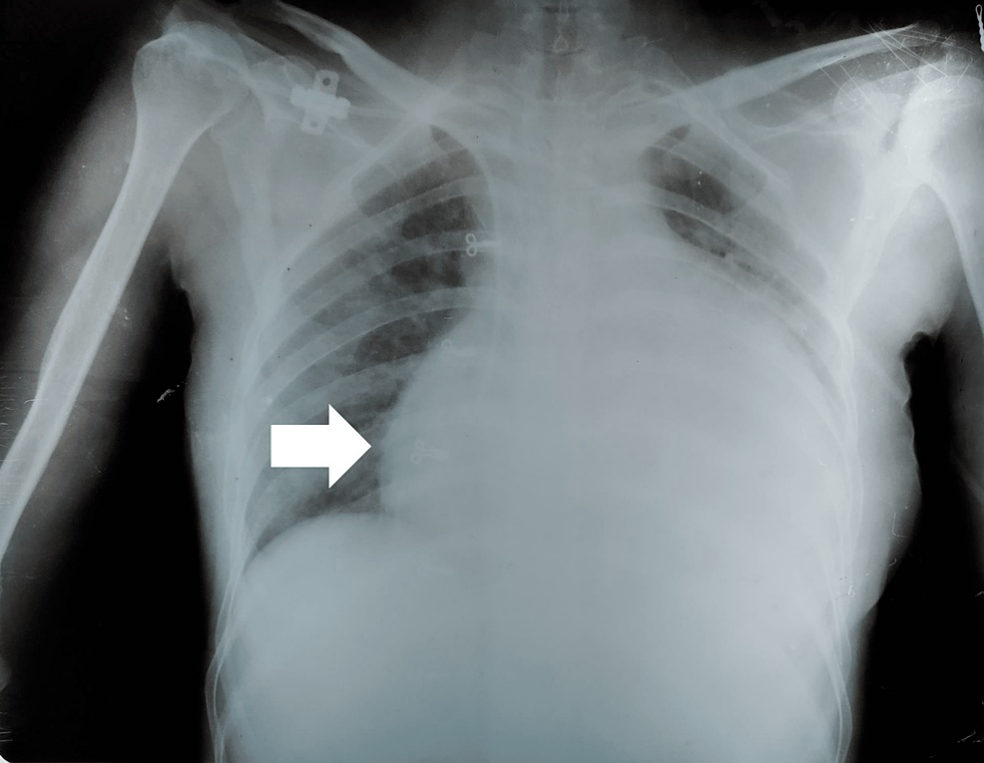
Plain chest X-ray of the patient showing enlarged cardiac silhouette (arrow)

Her chest X-ray (CXR) revealed an enlarged cardiac silhouette that raised the suspicion of either a massive pericardial effusion or a massive cardiomegaly (Figure 2). A 2D echocardiography confirmed that it was a massive pericardial effusion. Her pericardial fluid was sent for examination. The following test results were insignificant or inconclusive: Ziehl-Neelsen staining, adenosine deaminase (ADA), and gram staining. The cytopathology of the pericardial fluid indicated moderate cellularity with few polymorphs and macrophages in the background of an eosinophilic proteinaceous material and red blood cells. Her electrocardiography (ECG) findings revealed the presence of short-wave complexes.

**Figure 3:**
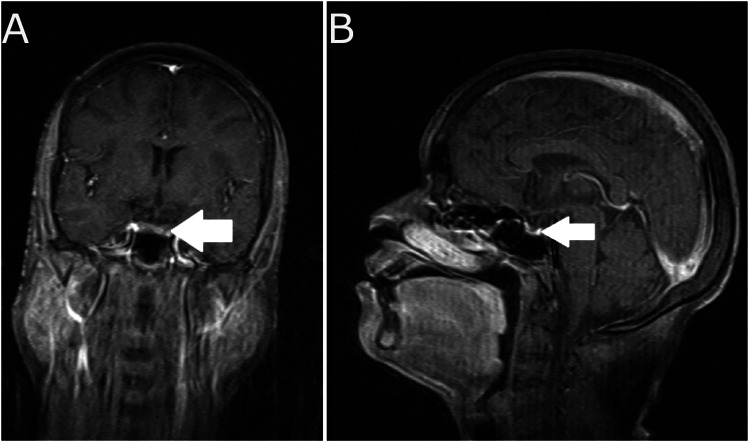
(A) T1 weighted MRI of the coronal section of the brain; (B) T1 weighted MRI of the sagittal section of the brain The scans reveal thinning of the pituitary gland with partially empty sella (arrows). However, the pituitary stalk is normal in length and width. MRI: magnetic resonance imaging

For further evaluation, a battery of endocrinological tests was ordered (Table 2). The hormone levels pointed towards adrenal insufficiency and hypopituitarism. A gadolinium-enhanced MRI of the brain was thus advised to the patient which revealed thinning of the pituitary gland (atrophic pituitary gland) with partially empty sella (Figure 3). Following the initial symptomatic management and after the diagnosis was made, she was started on appropriate hormone replacement to which she responded well.

**Table 2:** Results of hormonal investigations of the patient TSH: thyroid stimulating hormone, ACTH: adrenocorticotropic hormone, FSH: follicle stimulating hormone, LH: luteinizing hormone

Parameter	Value	Reference Range
Serum TSH, µIU/mL	0.63	0.35-5.5
Serum free T3, pg/mL	1.28	2.30-5.0
Serum free T4, pmol/mL	0.82	12-32
ACTH, pg/mL	<5	0-46
Prolactin, ng/mL	0.689	4.79-23.3
Serum Cortisol (morning), µg/dL	1.50	3.70-19.40
Serum FSH (post-menopausal), mIU/mL	4.82	23.00-116.30
Serum LH (post-menopausal), mIU/mL	1.89	5.16-61.99

## Discussion

The blood supply of the posterior pituitary comes from the inferior hypophyseal artery while the anterior pituitary is supplied indirectly by portal vessels coming from the hypothalamus and posterior pituitary. This low-pressure system feeding the anterior pituitary makes it more susceptible to ischemia after any vascular insult [5]. During pregnancy, this gland undergoes hyperplasia, increasing the nutritional and metabolic demands of the gland. In the case of a massive PPH, this gland undergoes ischemic necrosis in 1-2% of women who lose 1-2 L of blood with associated hypotension [3].

The clinical manifestations associated with this condition can result from selective loss of pituitary function or even panhypopituitarism. There is often a delay of months to years in the diagnosis of the condition due to the late presentation of vague and non-specific symptoms including fatigue, weakness, and anemia after the initial vascular insult [6]. Our patient presented six years after her last obstetric event because the signs of adenohypophyseal insufficiency are often delayed and subtle [7]. She gave birth to a stillborn and thus could not provide a history of failure to lactate. Owing to pituitary dysfunction and hypoprolactinemia, failure to lactate can occur along with a low serum level of prolactin as seen in this patient. Her amenorrhea and gradual deterioration after the delivery were, to the most extent, neglected until she was hospitalized.

In our patient, the history was also notable for six years of amenorrhea and low serum levels of follicle-stimulating hormone (FSH) and luteinizing hormone (LH) compared to the post-menopausal levels, indicating pituitary dysfunction as the cause of early menopause. Another less frequent cause of panhypopituitarism is lymphocytic hypophysitis, an autoimmune condition that needs to be ruled out in the diagnosis of Sheehan syndrome [8]. The low serum prolactin level in our patient along with a history of PPH makes Sheehan syndrome a more likely diagnosis.

Hypothyroidism from the deficiency of thyroid-stimulating hormone (TSH) secreted by the anterior pituitary is responsible for a majority of clinical manifestations in our patient. Fatigue, loss of appetite, edema, hair loss, and dry wrinkling skin seen here can be explained by the deranged thyroid profile. A major cause of pericardial effusion is hypothyroidism, which usually gets corrected upon achieving a euthyroid state [9]. Pituitary dysfunction leads to disruption of normal adrenocorticotropic hormone (ACTH)-cortisol axis seen as adrenocortical insufficiency. Common features of adrenal insufficiency like weight loss, anorexia, nausea, vomiting, lethargy, and fatigue along with skin pigmentation and loss of axillary and pubic hair have been observed in people with primary adrenal failure [10]. Hypotension and tachycardia accompanied by impaired consciousness at the time of admission of our patient indicate that she was in a state of adrenal crisis secondary to ACTH deficiency which was confirmed by low serum levels of ACTH and cortisol on analysis (Table 2).

According to a cohort study in Costa Rica, approximately 50% of patients with Sheehan syndrome develop panhypopituitarism, whereas only adrenal insufficiency is seen in around 33% of patients, and the remaining present with just hypothyroidism [11]. Confirmation of diagnosis of Sheehan syndrome is done by imaging of the pituitary gland and sella turcica where an empty sella is present in about 70% of patients, and a partially empty sella is present in about 30% of patients on MRI [12]. After the establishment of diagnosis, the aim of the treatment is to correct the endocrine imbalances such as hypoglycemia and adrenal insufficiency that warrant urgent care. The normal function of the thyroid, adrenals, and ovaries can be maintained by hormone substitution for life. It is important to have regular follow-ups for response assessment and dose regulation of the medications.

## Conclusions

Though the occurrence of Sheehan syndrome has slowly declined over time with better management of labor and delivery, in the resource-poor healthcare settings of developing countries, its cases are still witnessed owing to neglected blood loss during delivery and poor management. The late presentation of this condition can result in a delay in diagnosis. The condition can be fatal and warrants an early diagnosis through recognition of symptoms as well as a blood workup in a female with massive PPH.
